# Functional neuroimaging of fatty acid amide hydrolase inhibition in posttraumatic stress disorder: a randomized clinical trial

**DOI:** 10.1038/s41398-026-03864-3

**Published:** 2026-02-06

**Authors:** Ryann Tansey, Irene Perini, Gavin N. Petrie, Connor J. Haggarty, Raegan Mazurka, Adam Yngve, Sarah Mina, Madeleine R. Jones, Hilda Engelbrektsson, Andrea J. Capusan, Matthew N. Hill, Markus Heilig, Leah M. Mayo

**Affiliations:** 1 Department of Psychiatry, Cumming School of Medicine, University of Calgary, Calgary, AB Canada; 2 Hotchkiss Brain Institute, University of Calgary, Calgary, AB Canada; 3 Mathison Centre for Mental Health Research and Education, University of Calgary, Calgary, AB Canada; 4 Center for Social and Affective Neuroscience, Department of Biomedical and Clinical Sciences, Linköping University, Linköping, Sweden; 5 Center for Medical Image Science and Visualization (CMIV), Linköping, Sweden; 6 Department of Cell Biology and Anatomy, University of Calgary, Calgary, AB Canada

**Keywords:** Psychiatric disorders, Neuroscience

## Abstract

The endocannabinoid ligand anandamide (AEA) plays a role in fear extinction, the conceptual foundation of the gold standard treatment for posttraumatic stress disorder (PTSD), exposure-based psychotherapy. Converging evidence from animal models and non-clinical human studies highlights the potential to enhance fear extinction pharmacologically by inhibiting the AEA catabolic enzyme, fatty acid amide hydrolase (FAAH). However, in our randomized clinical trial (*n* = 100), a FAAH inhibitor did no better than placebo at enhancing the response to exposure-based therapy in PTSD. Here, we used functional magnetic resonance imaging to investigate the neurobiological effects of FAAH inhibitor treatment on resting-state functional connectivity and the neural correlates of emotional processing (*n* = 76 scanned). We found that greater symptom improvement was significantly related to lower functional connectivity between ventromedial prefrontal cortex (vmPFC) and right dorsolateral prefrontal cortex (dlPFC), as well as lower task activation of the right dlPFC. Further, we found that self-reported symptoms at the time of scan were associated with increased functional connectivity of the vmPFC and the amygdala across the cortex (mainly in the ventral attention network and sensorimotor network, respectively). However, while we confirmed that 4 weeks of FAAH inhibition significantly increased AEA, there were no significant differences in functional connectivity or task activation between treatment groups. These findings suggest that FAAH inhibition does not affect intrinsic functional connectivity or emotional task response in PTSD, and that the dlPFC may play an important role in the response to exposure-based psychotherapy.

## Introduction

Posttraumatic stress disorder (PTSD) is a serious psychiatric condition that can develop following exposure to traumatic events and is characterized by symptoms of hyperarousal, intrusive and distressing thoughts, avoidant behaviors, and negative affect [1]. Data from World Health Organization World Mental Health Surveys found that the aggregate risk of developing PTSD following trauma exposure was 4.0%, though this varies by trauma type [2]. The same survey found that the prevalence of trauma exposure was 70.4% [3]; when taken together, these statistics suggest that PTSD affects a very large number of individuals worldwide.

While there are a number of potential treatments for PTSD, substantial attention has been paid to those that are based on the mechanisms of fear extinction, as they have proven to be effective [4]. Exposure therapies, which are widely considered a gold-standard treatment, are thought to facilitate fear extinction by allowing the patient to develop an association between their trauma triggers and a safe context, which overrides the initial fear memory [4, 5]. Converging research from preclinical and human studies has identified the circuit between the amygdala and the ventromedial prefrontal cortex (vmPFC) as an important neural substrate of the fear extinction mechanism [6–10]. Dysregulated amygdala-vmPFC function has been proposed as a key etiological mechanism in PTSD [6, 11]. This circuit is also thought to be involved in a number of other functions, such as emotion regulation [12], which is altered in PTSD [13, 14]. The clinical importance of this shared circuit in PTSD is further demonstrated by evidence that treatment response to exposure therapy can be predicted based on brain activation in the amygdala and prefrontal cortex during emotional reactivity and regulation tasks [15]. This suggests that treatments that modulate the function of the amygdala-vmPFC circuit, which appears to be involved in both fear extinction and emotional regulation, may prove an effective clinical opportunity for PTSD.

Unfortunately, the dropout rate among patients with PTSD from guideline-recommended therapies is high (with one recent meta-analysis of clinical trials estimating an average rate of ~20%), and appears to be among the highest for trauma-focused therapies such as exposure therapy [16]. Enhancing the efficacy by decreasing the duration of therapies that are based on fear extinction could prove to be an important step towards increasing patient retention and addressing the mental health burden of trauma worldwide [17].

Over the past few decades, mounting translational evidence has emphasized the importance of the endocannabinoid (eCB) system in psychiatric disorders [18]. Stress- and trauma-based disorders, including PTSD, have been an area of particular interest [19]. The cannabinoid 1 (CB1) receptor is thought to confer much of the behavioral function attributed to both exogenous and endogenous cannabinoid compounds [20]. The naturally occurring eCB ligands are the full CB1 agonist 2-arachidonoylglycerol (2-AG) and the partial agonist *N*-arachidonoylethanolamine, also known as anandamide (AEA). Evidence from animal models has emphasized the important role of AEA in fear extinction [19], which positions it as an intriguing clinical opportunity for PTSD. Increased concentrations of endogenous AEA, whether through genetic manipulation [21] or pharmacological intervention [22, 23], result in enhanced extinction of conditioned fear memories. The effects of AEA on fear extinction appear to rely on projections from the medial prefrontal cortex (PFC) to the amygdala [24], further underscoring the clinical potential of AEA in PTSD.

Multiple translational studies conducted in human participants have confirmed the role of AEA in human fear extinction behaviors. Fatty acid amide hydrolase (FAAH) is the enzyme that degrades AEA, and genetically driven alterations in FAAH activity can produce changes in AEA concentrations in the brain and in the periphery. Individuals with a single nucleotide polymorphism that downregulates FAAH activity show both enhanced fear extinction and increased functional connectivity between the amygdala and the vmPFC [21, 25]. Studies using positron emission tomography to quantify FAAH in the amygdala found that lower FAAH (assumed to be indicative of higher levels of AEA) was associated with increased functional connectivity between the amygdala and the vmPFC [26] and attenuated responses to threatening stimuli in the amygdala [27]. Importantly, the effects of AEA on fear extinction can also be pharmacologically induced. The administration of a FAAH inhibitor (FAAHi), which can increase peripheral AEA approximately ten-fold, resulted in enhanced recall of fear extinction and decreased autonomic and emotional responses to stress in a non-clinical sample of adults [28]. Further, elevated AEA may confer resilience to the development of psychiatric symptoms, such as substance use disorder, following childhood maltreatment [29]. Given that AEA levels can enhance fear extinction, and appear to be related to function and connectivity in the amygdala-vmPFC circuit in both preclinical models and humans, we hypothesized that a FAAHi could potentially target not just the symptomatology of PTSD, but also its underlying neural circuits.

To this end, we ran a double-blind, randomized placebo-controlled clinical trial assessing the efficacy of the reversible FAAHi, JNJ-42165279, in combination with exposure-based psychotherapy in PTSD [30]. We found that the FAAHi was no more effective than placebo at decreasing PTSD symptoms across the 12-week trial. This result was unexpected, given the strong rationale behind the hypothesis. Here, we assess the effects of FAAHi on emotional processing and functional connectivity in the brain following 4-weeks of FAAHi or placebo treatment, to determine whether the lack of clinical effect is related to the underlying neurobiology.

## Materials and methods

### Trial design and interventions

**Fig. 1:**
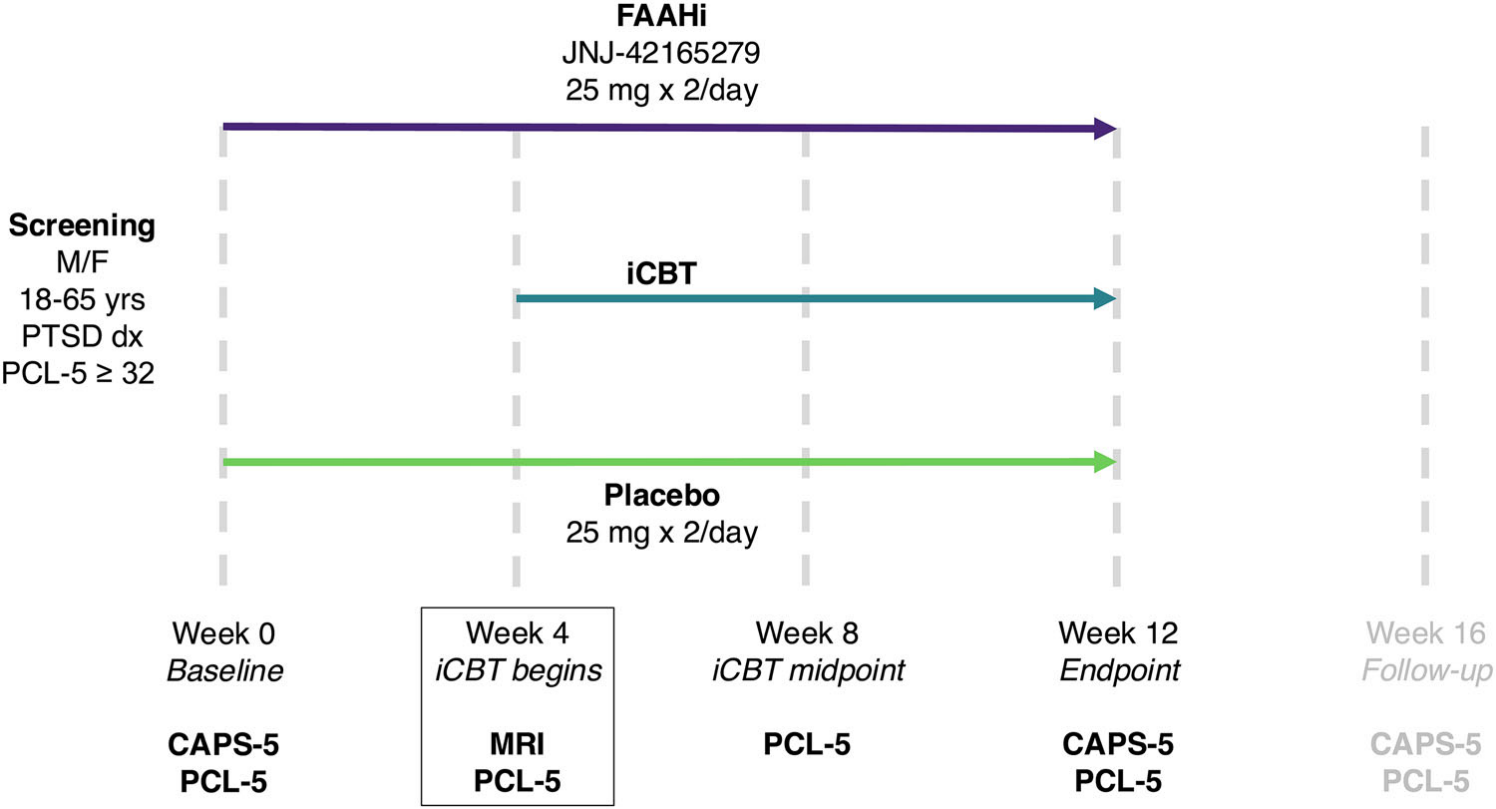
Clinical trial procedure schematic. Participants were randomized to either the FAAHi or the placebo groups at baseline (week 0). The pharmacological dosing regimen lasted for 12 weeks following baseline. After 4 weeks, participants began an internet-based CBT program (iCBT), which lasted another 8 weeks. The endpoint of the clinical trial, when both the FAAHi/placebo and the iCBT were finished, was 12 weeks after baseline. A phone follow-up appointment occurred 16 weeks following baseline. The data from the follow-up session are not included in this study.

The clinical trial (EudraCT 2020-001965-36) [30] ran from October 2020–December 2023. Regulatory approvals were obtained from the Swedish Ethical Review Authority (Dnr 2020-57043) and the Swedish Medical Products Agency. Participants were randomized at a 1:1 ratio (stratified by sex) to either the FAAHi (two 25 mg doses of FAAHi JNJ-42165279 daily) or placebo (two 25 mg doses of placebo) groups, and group assignment was double-blinded to both participants and researchers. Participants took the FAAHi or placebo for 12 weeks in total. After 4 weeks on either FAAHi or placebo, participants began an internet-delivered cognitive behavioural therapy (iCBT) program for PTSD which lasted a further 8-weeks. During this time, the participants continued to receive their FAAHi or placebo regimen. A schematic of study procedures can be seen in Fig. 1, and a detailed description of the visit timeline in the Supplementary Material. All clinical and eCB data has been reported previously [30]. The target sample size of *n* = 100 was based on a power calculation for a moderate effect size of *d* = 0.6 at a power of 85% [30]. Informed consent was obtained from all participants.

It is important to note that the neuroimaging session took place after 4 weeks on FAAHi or placebo, and thus prior to the beginning of iCBT. Both resting state and task fMRI data were collected. The task used in the scanner was the emotional conflict task [15, 31]. A previous study showed that neural activation during this task was able to predict PTSD patients’ treatment response to exposure therapy [15]. In order to validate this potential biomarker, we employed the same task in this study prior to the commencement of psychotherapy. This study design allowed for the assessment of neurobiological correlates of the pharmacological effects of the FAAHi without the confounding factor of the psychotherapy program.

### Participants

Participants were individuals aged 18–65 years with a clinical diagnosis of PTSD, based on the *Diagnostic and Statistical Manual of Mental Disorders-5* (DSM-5) [1], and were recruited at Linköping University in Linköping, Sweden. Participants were excluded if they had a lifetime history of any major psychiatric disorder, any current psychiatric condition, or substance use disorder. Included participants had to be able to identify an index trauma, and those with complex PTSD or ongoing trauma were excluded from the study. For detailed exclusion criteria, see the Supplementary Material. See the CONSORT chart in Supplementary Fig. S1 for a breakdown of the number of participants who were screened, enrolled, and included in each of the fMRI analyses, and see Table 1 for the demographics and baseline clinical characteristics in each analysis by treatment group. Supplementary Table S1 contains a summary of the adverse events that occurred during the clinical trial.

**Table 1:** Demographic and baseline clinical data stratified by group for both the task and resting state fMRI analyses. *P*-values that are significant at the level of <0.05 are in bold.

	Task final sample (*n* = 76)			Resting state final sample (*n* = 76)		
	FAAHi *n* = 40	Placebo *n* = 36	*p*-value	FAAHi *n* = 37	Placebo *n* = 39	*p*-value
Sex: female (%)	32 (80.0%)	33 (82.5%)	0.2640^a^	30 (81.1%)	36 (92.3%)	0.2680^a^
Age (years)	36.55	36.28	0.9179^b^	35.38	36.21	0.7441^b^
Occupation			0.2405^a^			0.2529^a^
Employed	21 (52.5%)	17 (47.2%)		19 (51.4%)	19 (48.7%)	
Studies	12 (30.0%)	7 (19.4%)		12 (32.4%)	8 (20.5%)	
Unemployed	7 (17.5%)	12 (33.3%)		6 (16.2%)	12 (30.8%)	
Current psychoactive medication (%)	34 (85.0%)	29 (80.6%)	0.7163^a^	31 (83.8%)	32 (82.0%)	0.6744^a^
Current psychiatric co-morbidity (%)^c^	25 (62.5%)	25 (69.4%)	0.6928^a^	22 (59.5%)	27 (69.2%)	0.5158^a^
PTSD diagnosis (CAPS-5)	33 (82.5%)	32 (88.9%)	0.6427^a^	30 (81.1%)	34 (87.2%)	0.6788^a^
PTSD severity score (CAPS-5)	31.15	33.92	0.1067^b^	30.46	33.54	0.0670^b^
Self-reported PTSD symptoms (PCL-5)^d^	43.32	46.53	0.2264^b^	42.75	45.85	0.2741^b^
Anxiety symptoms (CPRS-S-A)^d^	10.39	10.91	0.5834^b^	10.18	10.93	0.4247^b^
Depressive symptoms (CPRS-S-A)^d^	10.30	10.53	0.8101^b^	10.10	10.63	0.5775^b^
Sleep quality (PSQI)^d^	10.36	11.91	0.0821^b^	10.00	11.60	0.0655^b^
Number of censored volumes in fMRI scan	30.43	29.67	0.9457^b^	18.57	28.69	0.2843^b^
Plasma AEA concentration (µM)	0.7027	0.8903	**0.0280**^b^	0.6899	0.8726	**0.0291**^b^
Plasma 2-AG concentration (µM)	3.3106	2.8795	0.7582^b^	3.2530	3.3221	0.9838^b^

^a^*p*-values derived from a χ^2^ test of independence.

^b^*p*-values derived from a two independent sample *t*-test.

^c^Defined as any current co-morbid psychiatric disorder according to the MINI.

^d^Missing values for both the task and rest fMRI sub-samples. PCL-5: *n* = 1 from FAAHi; CPRS-S-A anxiety: *n* = 2 from placebo; CPRS-S-A depressive: *n* = 4 from placebo; and PSQI sleep quality: *n* = 4 from FAAHi and *n* = 4 from placebo. Means and *p*-values are calculated on the sample after removing the missing values. There are the same number of missing values for each measure for both the task and rest fMRI sub-samples.

### PTSD symptom measures

Both clinician-assessed and self-reported PTSD symptom severity measures were collected from participants. The clinician assessed symptoms were scored with the Clinician-Administered PTSD Scale for *DSM-5* (CAPS-5) [32]. The change in CAPS score between baseline and endpoint (endpoint CAPS score – baseline CAPS score; ΔCAPS) was used as the primary outcome for symptom improvement. A more negative ΔCAPS reflected a greater decrease in symptoms and an increased response to treatment. The self-reported symptoms were measured with the Posttraumatic Stress Disorder Checklist for *DSM-5* (PCL-5) [33], and provided a measure for the level of current PTSD symptoms at the time of the scan. No other PCL-5 scores from other time points were included in the analysis. For detailed information on these two measures, see the Supplementary Material [34, 35].

### Plasma eCB analysis

Plasma concentrations of endocannabinoids, including N-arachidonoylethanolamide (AEA) and 2-arachidonoylglycerol (2-AG) were quantified using mass spectrometry based on the method detailed by Qi et al. [36], and in the Supplementary Material.

### MRI data acquisition

MRI data were collected at the Center for Medical Image Science and Visualization (CMIV) at Linköping University, Sweden using a Siemens MAGNETOM Prisma 3-Tesla scanner (Siemens Healthcare AB, Stockholm, Sweden), with a 64-channel head coil. Specific scan parameters are detailed in the Supplementary Material.

### MRI preprocessing

All data was preprocessed using AFNI, version 23.1.02 [37]. For both task and resting-state scans, volumes with motion greater than 0.3 mm (enorm) were censored, along with volumes where more than 5% of the voxels were calculated to be outliers (outlier fraction of 0.05). Participants with 30% or more of their scan volumes censored were excluded from the analysis. Detailed information on the preprocessing pipelines for both the task and resting-state analyses can be found in the Supplementary Material [38, 39].

### Emotional conflict task

We used an in-scanner event-related design to assesses emotional conflict processing [31]. The task functions as an emotional Stroop task. Trials were congruent (same facial expression and word; e.g. a fearful face that says “FEAR”) or incongruent (different facial expression and word; e.g. a fearful face that says “HAPPY”). There were four different conditions of interest in the study: congruent trials that followed congruent trials; congruent trials that followed incongruent trials; incongruent trials that followed congruent trials; and incongruent trials that followed incongruent trials. In the original study [31], incongruent trials that followed congruent trials were interpreted as engaging emotional conflict monitoring processes, while incongruent trials that followed incongruent trials were thought to recruit emotional conflict regulation. Increased activation in the vmPFC and ventral striatum during the emotional conflict regulation condition of this task at baseline has been previously shown to predict better treatment outcomes in individuals with PTSD [15]. For a detailed task description and specific in-scanner presentation parameters, see the Supplementary Material.

#### Behavioral data analysis

Accuracy and reaction time effects were evaluated using linear mixed effects models implemented with the packages *lme4* [40] and *lmerTest* [41] in R (version 4.3.1). We utilized linear mixed-effects models so that we could better account for the within-subject repeated measures structure of the data, while also controlling for other potential confounding variables. In line with the previous literature, we included current trial, previous trial, and a current trial x previous trial interaction in the models in order to detect the emotional conflict resolution effect [31]. All models also included sex as a between-subjects covariate of no interest. Subject was included as a random intercept (1|Subject) in all models to account for repeated measures within individuals. We did not include random intercepts that accounted for the variation in the intercepts among current trial and previous trial within subjects (1|Subject:Current Trial or 1|Subject:Previous Trial) as the model with this random effects specification was determined to be singular for the accuracy and reaction time data.

#### fMRI data analysis

Task fMRI data were analyzed at the group level using the AFNI program *3dLMEr* [42], in AFNI version 23.1.02. Linear mixed effects models were specified as follows:

$$\begin{array}{l}X+\mathrm{Previous}\mathrm{Trial}* \mathrm{Current}\mathrm{Trial}+\mathrm{Sex}+\mathrm{Motion}+(1|\mathrm{Subject})\\ +(1|\mathrm{Subject}:\mathrm{Previous}\mathrm{Trial})+(1|\mathrm{Subject}:\mathrm{Current}\mathrm{Trial})\end{array}$$

where *X* denotes the covariate(s) of interest (treatment group, AEA levels, PCL-5 scores, or ΔCAPS) and Motion is defined as the number of censored volumes per condition. Significant cluster size was determined using a voxelwise threshold of *p* = 0.002 [43] and cluster forming threshold of α = 0.05, corresponding to a cluster size of 13 voxels, which was calculated using AFNI functions *3dFWHMx* (to estimate the smoothness of the task data error time series) and *3dClustSim*. These simulations were run based on a grey matter mask of the union of EPI-masks of participants surviving censoring, which was subsequently multiplied with the MNI grey matter mask to restrict the number of voxels used for statistical testing to the grey matter. *Post-hoc* contrasts were specified in the *3dLMEr* model.

### Resting state analysis

#### Region of interest analysis

First, we conducted two region of interest (ROI) analyses based on a priori hypotheses: that the FAAHi group would have increased connectivity compared to placebo between the amygdala and the vmPFC, and increased anticorrelation (more negative correlation) between the anterior insula and the vmPFC. We hypothesized that we would see increased amygdala-vmPFC connectivity because dysregulation of the amygdala in PTSD is thought to stem in part from a decreased top-down influence from the vmPFC [6, 44, 45], and previous literature has found that healthy individuals with a genetic variation that downregulates FAAH activity had increased connectivity between these areas compared to controls [21]. The anterior insula-vmPFC hypothesis was based on a study that found that resilience following childhood maltreatment may be associated with increased AEA and more anticorrelated (negative) connectivity between the anterior insula and the vmPFC compared to controls in adulthood [29]. We therefore hypothesized that increasing AEA pharmacologically in our sample with a history of trauma would result in more strongly anticorrelated connectivity between the two areas.

The vmPFC ROI was defined using the Harvard-Oxford cortical structural atlas [46], as was the ROI for the bilateral amygdala [47–50]. A parcellation of the insula that was formed from combining multimodal imaging data (functional and structural) was used to derive our bilateral anterior insula ROI (we used the coarsest parcellation with a clustering level of *k* = 2) [51]. We used bilateral ROIs for the vmPFC, amygdala, and insula, because we did not have any prior hypotheses regarding specific areas of these regions or hemispheres in which we would see an effect, and we wanted to limit the number of statistical tests performed in order to decrease the chances of Type I error.

The average BOLD time course was extracted from each of the three ROIs using AFNI’s *3dmaskave* program. The functional connectivity between ROIs was calculated by applying a Pearson correlation to the two time courses of interest (vmPFC-amygdala, and vmPFC-anterior insula). Group-level statistics were conducted using linear models in R (the *lm* function). Sex and motion (number of censored volumes) were included as nuisance covariates in all linear models. Separate models were created to investigate each covariate of interest (treatment group, AEA levels, PCL scores, ΔCAPS scores). The Benjamini-Hochberg procedure was used to calculate false discovery rate in order to correct for multiple comparisons [52].

#### Exploratory whole brain analysis

The vmPFC and amygdala were used as seeds for the whole-brain analysis (defined using the same parcellations as in the ROI analysis). The average time courses in these regions were extracted using *3dmaskave*, and voxelwise functional connectivity (correlation to the seed region’s average time course) was calculated using the AFNI program *3dTcorr1D*. Group-level analysis was conducted using *3dMVM* [53]. The model specification was similar to the ROI analysis (sex and motion as nuisance covariates, and different models for each of the four covariates of interest). *3dFWHMx* was used to estimate the smoothness of the error time series of the resting state data, and the resultant autocorrelation function parameters were input into *3dClustSim* to estimate cluster thresholds. Significant cluster size was determined using a voxelwise threshold of *p* = 0.002 and cluster forming threshold of α = 0.05 [43], corresponding to a cluster size of 11 voxels.

## Results

### Sample demographics and symptom improvement

The main demographic and clinical findings from the trial can be found in [30]. Briefly, participants improved in symptoms over time but there was no difference between treatment groups (Table 1). Except for one participant with an extreme value (> 3 standard deviations from the mean) for their AEA measure at time of scan, all participants who were included in the PCL-5 analysis were included in the AEA analysis. Figures depicting the levels of AEA (Supplementary Fig. S2), CAPS scores (Supplementary Fig. S3), and PCL scores (Supplementary Fig. S4) over the course of the clinical trial can be found in the Supplementary Material. More detailed information regarding the types of index trauma in the sample is reported elsewhere [30] and in Supplementary Table S2.

### Failure to replicate the primary effects of the emotional conflict task in a sample of mostly female PTSD patients

As the previous literature is mixed as to whether the behavioral effects of the emotional conflict task can be replicated in populations with trauma exposure [54–56], we first determined if it could be detected in our sample. While we were unable to replicate the behavioral conflict resolution effect on reaction time or accuracy (Supplementary Fig. S5), we found a significant previous trial x current trial interaction in the fMRI data in 9 different clusters (Supplementary Table S3**;** Supplementary Fig. S6), though none of the significant clusters were in the rostral anterior cingulate cortex (ACC) [31]. For detailed behavioral and fMRI results, see the Supplementary Material.

### Individuals with higher current PTSD symptoms show greater functional connectivity to both the vmPFC and the amygdala throughout the brain

**Fig. 2:**
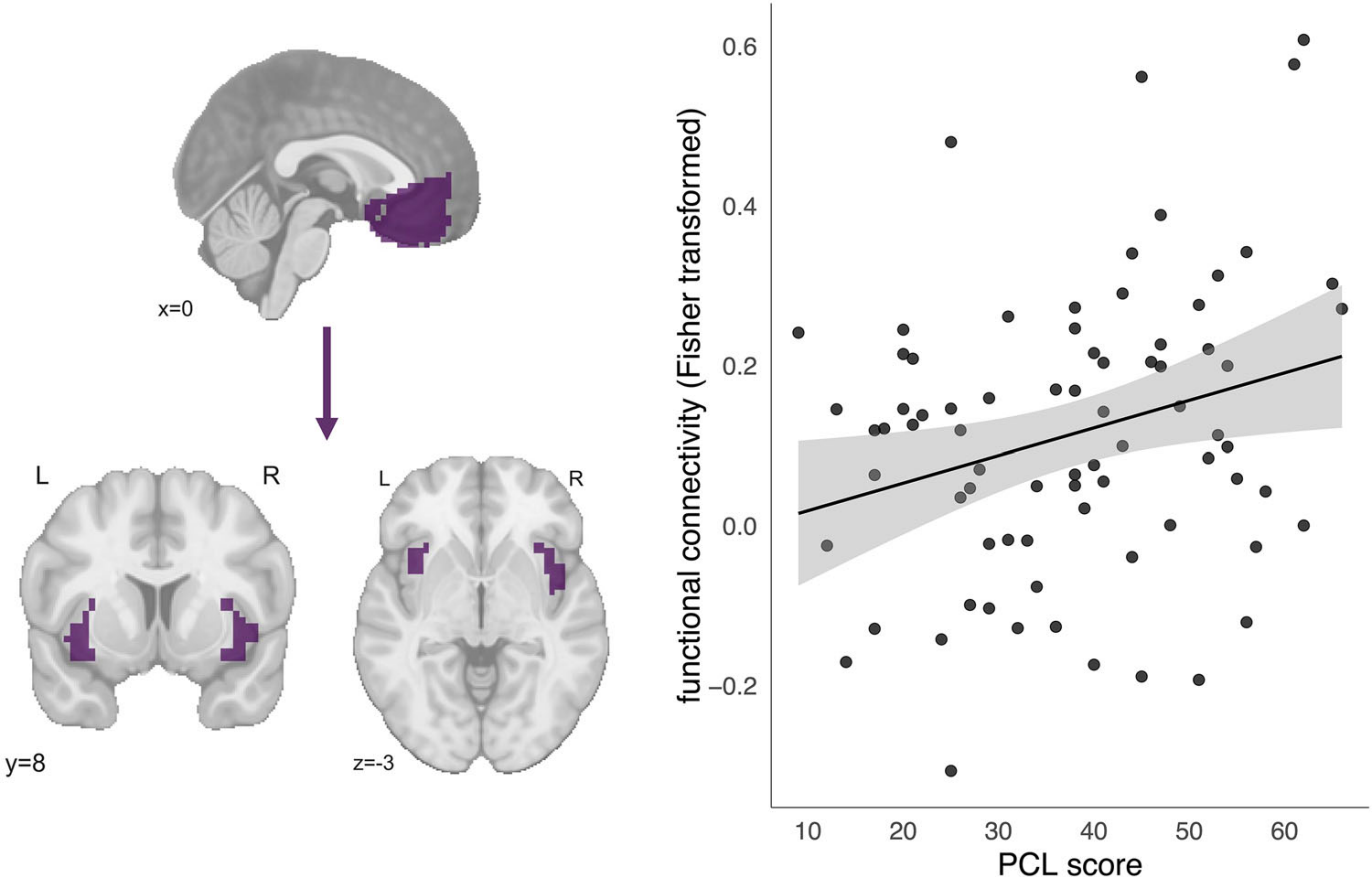
vmPFC-anterior insula connectivity is positively associated with self-reported PTSD symptoms. Functional connectivity units (on the *y*-axis) are Fisher-transformed correlations. Scatterplot trend line added for visualization purposes.

In the ROI analysis, there was no significant association between PCL symptom severity scores and amygdala-vmPFC connectivity (model *F*_3,72_ = 1.547, *p* = 0.210; PCL *β* = -0.001, PCL *t*_72_ = −0.876, PCL *p* = 0.384). Anterior insula-vmPFC functional connectivity was significantly associated with week 4 PCL symptoms, such that individuals who had greater self-reported symptoms at the time of the scan had greater connectivity between the two ROIs (Fig. 2; model *F*_3,72_ = 5.329, *p* = 0.002; PCL *β* = 0.003, PCL *t*_72_ = 2.498, PCL *p* = 0.0148), though this association was only trending following multiple comparisons correction (adjusted *p* = 0.0592). The residuals of the model met the assumptions of normality, heteroscedasticity, and independence.

Treatment group, AEA levels, and ΔCAPS scores were not significantly associated with amygdala-vmPFC connectivity or anterior insula-vmPFC connectivity. Variances of anterior insula-vmPFC and amygdala-vmPFC connectivity were similar across treatment groups (anterior insula-vmPFC: s_FAAHi_ = 0.030, s_PBO_ = 0.036, *F*_36,38_ = 0.812, *p* = 0.533; amygdala-vmPFC: s_FAAHi_ = 0.025, s_PBO_ = 0.025, *F*_36,38_ = 1.014, *p* = 0.965). There was a trend-level association between week 4 plasma AEA levels and anterior insula-vmPFC connectivity (model *F*_3,71_ = 4.514, *p* = 0.006; AEA *β* = −0.011, AEA *t*_71_ = −1.676, AEA *p* = 0.098, adjusted *p* = 0.1312).

**Fig. 3:**
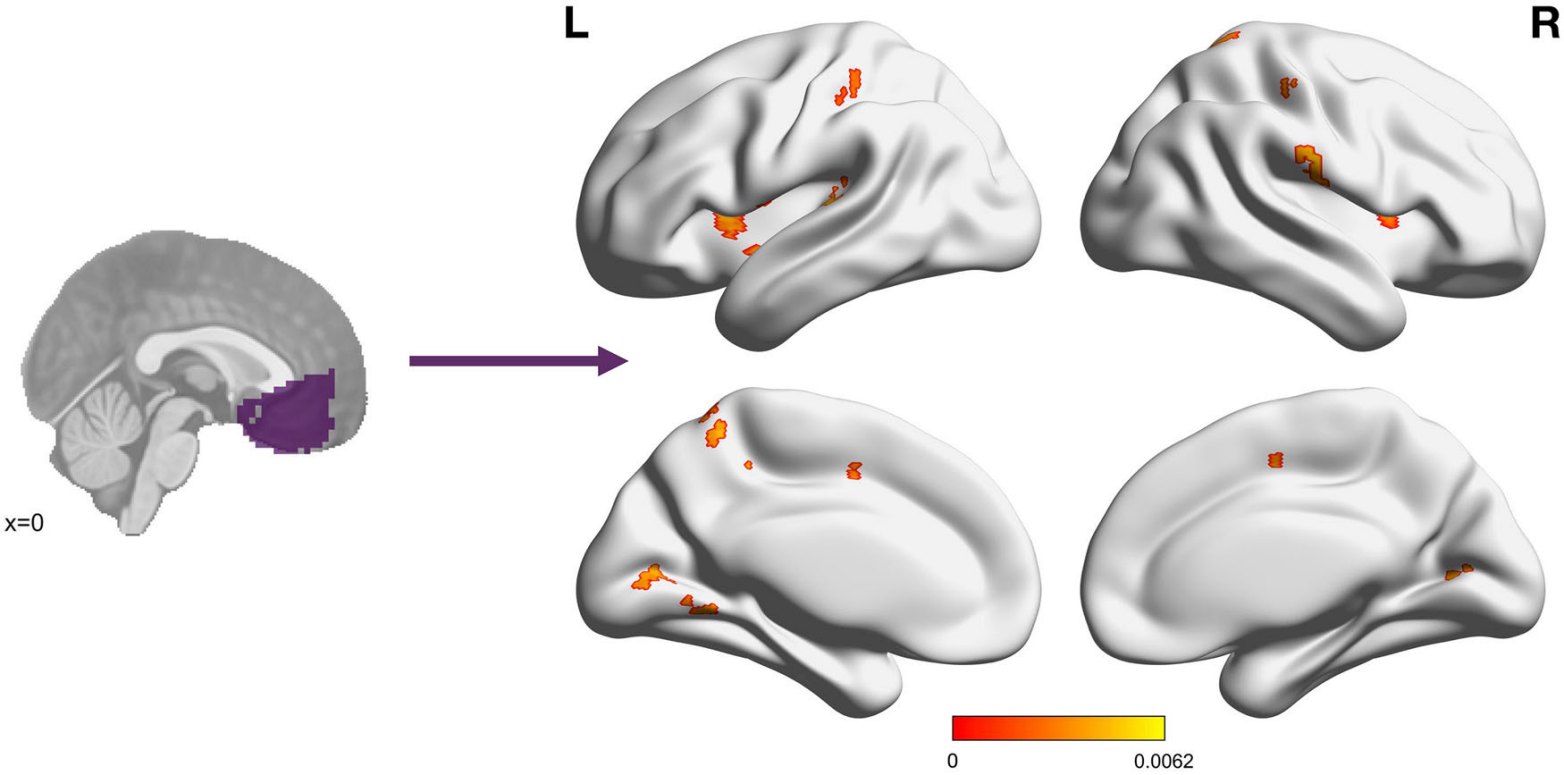
Self-reported PTSD symptoms positively associated with connectivity to the vmPFC in multiple clusters throughout the brain. Color bar depicts standardized *β* values for the effect of week 4 PCL score in a model defined as PCL + Sex + Motion using AFNI’s *3dMVM*. Significant cluster size was determined using a voxelwise threshold of *p* = 0.002 and cluster forming threshold of α = 0.05, corresponding to a cluster size of 11 voxels. Significant clusters were located in the bilateral anterior insula, postcentral gyrus, Rolandic operculum, and cuneus/primary visual cortex, right supramarginal gyrus, superior parietal lobule, supplementary motor area, claustrum, and thalamus, and left posterior insula, precuneus, middle cingulate cortex, auditory cortex, and cerebellum.

For the exploratory whole-brain analysis, there were 18 clusters throughout the brain where PCL scores were significantly associated with functional connectivity to the vmPFC (Fig. 3; Supplementary Table S4). For all clusters, the relationship was positive, such that *increased* functional connectivity to the vmPFC was associated with *higher* PCL scores. The majority of significant voxels overlapped with the ventral attention network (VAN), followed by the sensorimotor, the dorsal attention (DAN), and visual networks (as defined by the Yeo 7-network parcellation [57]).

**Fig. 4:**
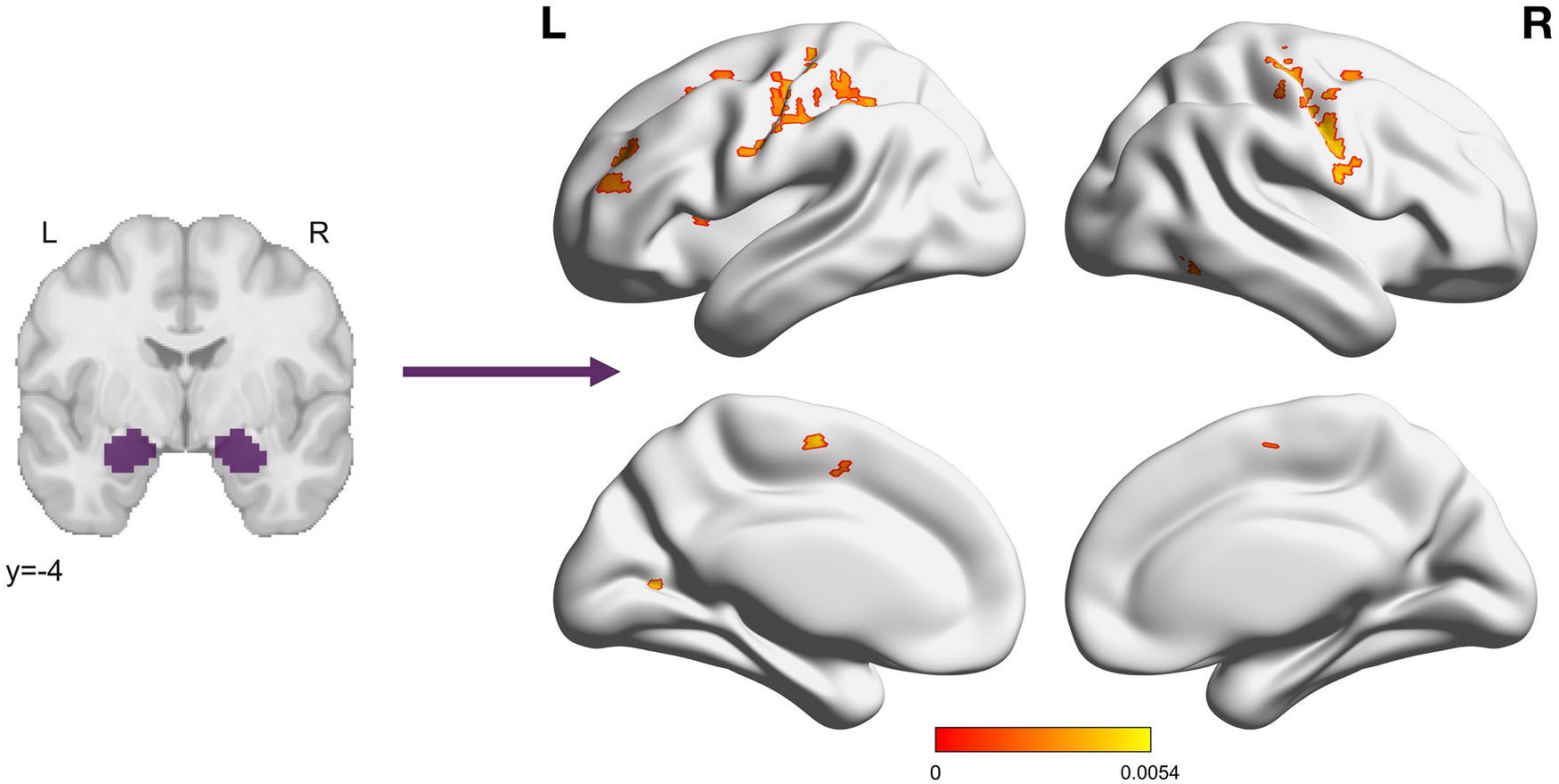
Self-reported PTSD symptoms positively associated with connectivity to the amygdala in multiple clusters throughout the brain. Color bar depicts standardized *β* values for the effect of week 4 PCL score in a model defined as PCL + Sex + Motion using AFNI’s *3dMVM*. Significant cluster size was determined using a voxelwise threshold of *p* = 0.002 and cluster forming threshold of α = 0.05, corresponding to a cluster size of 11 voxels. Significant clusters were located in the bilateral postcentral gyrus and Rolandic operculum, left precentral gyrus, intraparietal sulcus, premotor cortex, rostral middle frontal gyrus, anterior insula, supplementary motor area, dorsolateral prefrontal cortex, frontal eye field, and primary visual cortex, and the right fusiform face area.

There were also several clusters throughout the brain where PCL scores were associated with functional connectivity to the amygdala. In 14 clusters, PCL scores were positively associated with functional connectivity to the amygdala, such that individuals with *higher* PCL scores also had *increased* amygdala connectivity (Fig. 4; Supplementary Table S5). The majority of significant voxels overlapped with the sensorimotor network, followed by the DAN, VAN, frontoparietal, and visual networks.

### Greater symptom improvement is associated with less dlPFC activation during the task and less dlPFC-vmPFC functional connectivity at rest

**Fig. 5:**
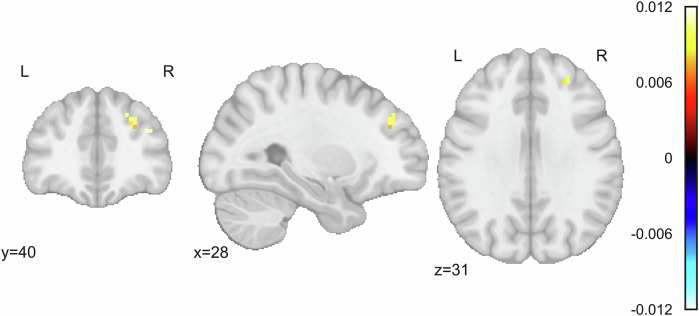
Change in clinician assessed PTSD symptoms between baseline and endpoint is significantly associated with activation in the right dlPFC during the emotional conflict task. Color bars depict standardized *β* values for the main effect of ΔCAPS in a linear mixed effects model defined as ΔCAPS + Previous Trial*Current Trial + Sex + Motion + (1|Subject) + (1|Subject:Previous Trial) + (1|Subject:Current Trial) using AFNI’s *3dLMEr*. Significant cluster size was determined using a voxelwise threshold of *p* = 0.002 and cluster forming threshold of α = 0.05, corresponding to a cluster size of 13 voxels. ΔCAPS was defined as endpoint CAPS – baseline CAPS.

In the whole-brain task analysis, there was a cluster in the right dorsolateral prefrontal cortex (dlPFC) with a significant main effect of ΔCAPS, where less activation during the task (across all conditions) was associated with more symptom improvement (more negative ΔCAPS; Fig. 5; cluster size of 30 voxels; peak MNI coordinates [28, 43]; peak standardized *β* = 0.012; peak *χ*^2^_2_ = 14.923; peak *Z*-score = 3.623). Treatment group, week 4 AEA levels, and week 4 PCL scores were not significantly associated with activation during the task.

**Fig. 6:**
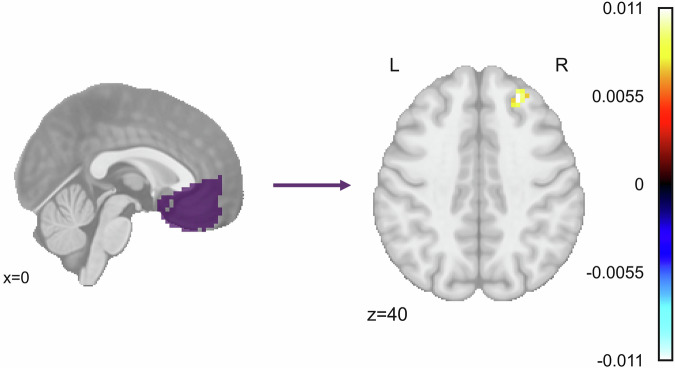
Change in clinician assessed PTSD symptoms between baseline and endpoint is significantly associated with functional connectivity between the vmPFC and the right dlPFC. Color bar depicts standardized *β* values for the effect of ΔCAPS in a model defined as ΔCAPS + Sex + Motion using AFNI’s *3dMVM*. ΔCAPS was defined as endpoint CAPS – baseline CAPS. Significant cluster size was determined using a voxelwise threshold of p = 0.002 and cluster forming threshold of α = 0.05, corresponding to a cluster size of 11 voxels.

In the whole-brain analysis of the resting state data, there was a cluster also in the right dlPFC where ΔCAPS was significantly and positively associated with functional connectivity to the vmPFC. Similarly to the task data, individuals with a greater decrease in CAPS score (and therefore, higher symptom improvement) had less vmPFC-dlPFC connectivity (Fig. 6b; cluster size of 34 voxels; peak MNI coordinates [25, 37, 40]; peak standardized *β* = 0.011; peak *F*_1,64_ = 30.598; peak *t*_64_ = 5.532). There were no significant clusters for treatment group or AEA levels, and no significant clusters for CAPS score, treatment group, or AEA levels for the whole-brain amygdala connectivity.

## Discussion

Here, we aimed to explore how elevating AEA via FAAHi impacts neural activity at rest and during emotion conflict processing in patients with PTSD. The results from this study are unexpected in two main respects. Firstly, there was no measurable effect of FAAHi on either clinical outcome [30] or neuroimaging indices (reported here), even though we verified FAAHi-induced elevations in AEA. Here, we found no significant differences between FAAHi and placebo groups in the BOLD response during emotional conflict processing or resting state functional connectivity. Secondly, the associations between both clinician- and self-reported PTSD symptoms and functional neuroimaging measures had opposite directionality compared to what could be expected based on previous literature. Presently, we will discuss the potential explanations for these findings, and their implications for future research into treatments for PTSD.

Contrary to our hypotheses, FAAH inhibition did not improve symptoms above and beyond the iCBT alone. We also did not find any relationship between treatment group or AEA concentrations in the fMRI data. A number of recent studies have added more nuance to the relationship between AEA and trauma, which may help to contextualize our results. A recent meta-analysis found that baseline AEA concentrations are increased in patients with stress-related disorders, and did not detect any stress-induced changes in AEA in patients either [58]. A preliminary study showed that adults with PTSD may have higher AEA plasma concentrations than controls [59] and a recent study of youth with high trauma burden found a positive association between peripheral AEA and PTSD symptom severity [60]. Further, a recent study found that baseline levels of AEA were not associated with treatment response in a sample of combat veterans with PTSD [61], which could explain why we did not see greater or more accelerated responses to iCBT in our FAAHi group compared to placebo. The involvement of the eCB system in trauma-related disorders appears to be complex, and necessitates future research.

Our neuroimaging findings were different from what would be expected based on previous PTSD literature. Firstly, there was no significant relationship between vmPFC-amygdala functional connectivity and CAPS-5 or PCL-5 measures. However, there was a significant, positive relationship between vmPFC-anterior insula functional connectivity and current PCL-5 scores. We recently demonstrated that individuals who had experienced childhood maltreatment and did not subsequently develop a substance use disorder showed low or negative functional connectivity between these two areas, which suggests that this may be a potential marker of resilience in a trauma-exposed population [29]. In the current study, we found that individuals with negative or close to 0 correlations between vmPFC and anterior insula activity had lower PCL-5 scores than those who had stronger, positive connectivity between these two areas. While not conclusive, when taken together with the findings from individuals with childhood maltreatment [29], our current findings point to a potential role of the vmPFC-anterior insula connection in resilience against mental health symptoms.

We also found that higher PCL-5 scores at time of scan were significantly associated with increased functional connectivity from both the vmPFC and the amygdala throughout widespread areas of the cortex. This was unexpected, as the vmPFC and the amygdala are thought to have opposing functions, with the vmPFC providing top-down inhibition on the amygdala [62, 63]. However, while the majority of significant clusters for the vmPFC were found in the VAN, most of the significant voxels for the amygdala were found in the sensorimotor network. This suggests that the mechanisms through which the increased vmPFC and amygdala connectivity are detrimental are complex and dependent on the function of the target regions. A study that was conducted in a sample of non-clinical women found that there was increased connectivity between the vmPFC and the VAN during a psychological stress task [64], which they interpret as indicative of increased regulation by the vmPFC over the bottom-up stress response. Another study found that healthy volunteers with greater connectivity between the amygdala and sensorimotor areas showed increased pain facilitation when viewing negatively valanced images [65], pointing to a potential role for amygdala-sensorimotor coupling in the emotional modulation of stressful experiences. It has also been postulated that sensory integration is an important aspect of the clinical response to trauma in PTSD [66]. Taken together, this suggests that there may be increased vmPFC-VAN and amygdala-sensorimotor connectivity in acute stressful experiences, which could explain why individuals with greater PTSD symptoms also show these functional connectivity patterns.

Finally, we did not see an association between vmPFC or amygdala task activation with treatment outcome. Instead, we found that both greater right dlPFC activation during the emotional conflict task and functional connectivity to the vmPFC were associated with less symptom improvement, as measured by the change in CAPS score between baseline and endpoint. This was unexpected both in the location of our results, and the directionality. However, other studies have implicated the dlPFC in emotion regulation function [67–69], which could account for the observed relationship in this location between change in CAPS and activation during the emotional conflict task. While less attention has been paid to the role of the dlPFC compared to the vmPFC-amygdala circuit in the previous PTSD literature, there has been consistent evidence that it is involved in the disorder. Whole-brain connectivity to the dlPFC was lower in PTSD patients compared to controls [70]. PTSD patients also had greater anticorrelation between the dlPFC and the precuneus compared to controls, and this was also associated with dimensional symptom severity [71]. Further, previous studies have shown that greater dlPFC activation during emotional processing of negative stimuli is related to decreased PTSD symptoms [72–74]. These findings would suggest that an increase in dlPFC activity may be beneficial in PTSD. In our current study, however, we found the opposite.

There are a few factors that could contribute to the surprising directionality of our results. The demographics of the sample in this study are markedly different than the historically typical samples in PTSD research, which have often been drawn from military veterans with an overrepresentation of males [75]. Notably, we had a sample that skewed female (85%). PTSD symptoms and emotional dysregulation have been shown to vary depending on both gender and trauma type [76, 77]. A meta-analysis found separable neural signatures for combat-related trauma and sexual/physical trauma [78]. Gender differences in PTSD have also been observed in functional connectivity [79] and activation during extinction recall [80], fear conditioning [81], and response inhibition [82]. These disparities in sample demographics could contribute to the differences that were found in this study compared to the previous literature.

This study has a number of strengths, including the relatively large sample size for a clinical population with neuroimaging, and the enriched number of females. However, there are limitations which should be noted. Mainly, we were only able to collect one MRI scan across the protocol duration. This was scheduled after 4 weeks on FAAHi but before the commencement of the psychotherapy. Longitudinal or semi-longitudinal studies carried out in individuals with PTSD throughout the dosing period would ameliorate this issue. Further, this study was restricted to individuals without complex PTSD. The findings presented may not be generalizable to individuals who cannot identify an index trauma, or who experience ongoing traumatization. This may also impact in our inability to detect any differences between treatment groups, as the symptom burden in our sample was not severe. Finally, the in-scanner task used for this study was chosen because it has been previously demonstrated to be predictive of treatment response to exposure therapy in PTSD [15]. However, a more recent meta-analysis found that replicability of this emotional conflict resolution effect is low, which could have limited our ability to detect any differences in neural activation between the FAAHi and placebo groups [83]. Future neuroimaging research on eCB signaling in patients with PTSD may benefit from the use of other task paradigms in the scanner, such as those measuring fear extinction directly [10]. Similarly, the effect estimates of variables such as PCL and ΔCAPS on BOLD activation were low, which limits the potential clinical translatability of our findings.

In sum, we demonstrate that FAAH inhibition did not have an effect on brain function in PTSD, for either emotional conflict processing or intrinsic functional connectivity. Taken together, our work suggests that enhancing AEA signaling in PTSD does not have an effect on emotional conflict processing or resting state functional connectivity in the brain, and that there may be important heterogeneity in brain function to consider when studying PTSD in diverse populations.

## Supplementary information

Supplemental Materials

## Data Availability

Code used in the generation of the results can be found at https://github.com/PaCT-Lab/Open/tree/main/MRI/PTSD-FAAHi.
