# Supplementary material for: Functional neuroimaging of fatty acid amide hydrolase inhibition in posttraumatic stress disorder: a randomized clinical trial
Source: Transl Psychiatry. 2026 Feb 6;16:95. doi: 10.1038/s41398-026-03864-3 (PMC12923879; doi:10.1038/s41398-026-03864-3)
Supplement: Supplementary file 1 — Supplemental Materials [file 41398_2026_3864_MOESM1_ESM.docx]

**Supplementary Material**

Tansey et al. *Functional neuroimaging of fatty acid amide hydrolase inhibition in posttraumatic stress disorder: A randomized clinical trial*

**1. Supplementary methods**

***Study overview***

After being deemed eligible, participants completed a total of 6 visits to the laboratory: a baseline visit prior to beginning the FAAHi or placebo (Visit 1; week 0); a MRI session where neuroimaging data was collected (Visit 2; weeks 3-4) and two laboratory sessions that took place after 3-4 weeks on FAAHi or placebo (Visits 3 and 4; weeks 3-4); a mid-therapy visit after 8 weeks on FAAHi or placebo (Visit 5; week 8; 4 weeks after commencing the therapy component); and a final post-treatment assessment which occurred at the end of the 12 weeks (Visit 6; week 12). There was also a follow-up by phone or internet which occurred 4 weeks following Visit 6. Clinician assessment of PTSD symptoms occurred at Visit 1, Visit 6, and during the phone follow-up. Self-report symptoms of PTSD and plasma eCB levels were collected at Visits 1, 3, 5, 6. Self-reported symptoms were also collected during the follow-up session.

***Participants***

Participants were excluded if they met any of the following criteria: a lifetime history of any major psychiatric disorder (e.g. schizophrenia, bipolar disorder); a current psychiatric condition (e.g. psychotic depression, severe eating disorder, borderline personality disorder, severe obsessive-compulsive disorder); cognitive impairment that could interfere with ability to give informed consent or comply with study procedures; a current clinical diagnosis of severe alcohol use disorder (but not mild or moderate), any other severe substance use disorder other than nicotine, or alcohol withdrawal syndrome; current or recent (within the last 6 months) clinically significant suicidal ideation or behaviors; insufficient memory of the trauma, or ongoing traumatization; dissociative disorder; ongoing treatment for PTSD; use of medications or foods that could interfere with the FAAHi or treatment; abnormal ECG results, or a history of other serious medical conditions.

***PTSD symptom measures***

The CAPS-5 consists of a structured clinical interview that assesses the severity of PTSD criteria symptoms, as well as other related symptomatology such as dissociation, distress, and impairment. For a diagnosis of PTSD, the individual must endorse at least one item in each of the criterion categories of re-experiencing symptoms, avoidance, negative cognitive and mood alterations, and alterations of arousal and reactivity, with symptoms lasting for at least a month, and causing impairment of regular functioning. The CAPS-5 also can provide an assessment of improvement compared to a previous time point. Here, we used the difference in CAPS-5 (ΔCAPS) scores from baseline prior to beginning any treatment to the end of the 12 weeks of the clinical trial, i.e., the conclusion of both the pharmacological intervention and the iCBT, to measure symptom change. A higher response to treatment is indicated by a ΔCAPS of a greater negative magnitude. A difference in scores of approximately 12-13 points or more between two time points is thought to be representative of reliable clinical change [1].

The PCL-5 consists of a questionnaire containing 20 5-point Likert scale items subjectively assessing both primary and related symptoms of PTSD. Scores can range from 0 (no PTSD symptoms) to 80 (maximum rating of symptoms). While there is variation in the literature, and diagnostic cut-off scores likely vary depending on population, scores of at least 31-33 are generally considered indicative of PTSD [2], and reliable clinical change is indicated by a difference in scores of approximately 15-18 [1].

***Plasma eCB analysis***

200 µL of plasma was mixed with 2 mL of acetonitrile containing 5 nM of d8-2-AG, 5 pM of d8-AEA, 40 pM of d4-PEA, and 40 pM of d4-OEA. The mixture underwent sonication for 30 minutes in an ice bath, followed by overnight incubation at -20°C to precipitate proteins. On the next day, the samples were centrifuged at 1500 × g to remove particulates, and the supernatant was collected into a clean glass tube. This supernatant was evaporated under a nitrogen stream and then washed with 250 µL of acetonitrile to extract the lipids adhering to the glass surface. After another round of nitrogen evaporation, the samples were reconstituted in 200 µL of acetonitrile and stored at -80°C until they were ready for liquid chromatography-mass spectrometry analysis.

***MRI data acquisition***

Anatomical, functional task, and functional resting state scans were all collected in the same session. For the emotional conflict task, a total of 825 volumes were acquired; for the resting state scan, 800 volumes were acquired. T1 anatomical scans were collected using a 3D MPRAGE sequence with the following parameters: TR = 2300 ms; TE = 2.36 ms; voxel size of 0.9mm^3^; flip angle = 8^o^; field of view of size 263 mm x 350 mm x 350 mm; no slice gap; with a total of 208 sagittal slices collected. Both functional scans were collected using an echoplanar imaging (EPI) sequence with the following parameters: TR = 878 ms; TE = 24 ms; voxel size of 3.0 mm isotropic; flip angle = 56^o^; field of view size 204 mm x 204 mm x 135 mm; no slice gap; with a total of 45 axial slices collected, autoaligned at T > C -20 degrees; and a multiband acceleration factor of 3.

***MRI preprocessing***

**Task fMRI**. The emotional conflict task data was preprocessed using a pipeline based on the suggested specifications for task fMRI data using the afni_proc.py program (example 6b on the afni_proc.py documentation). The EPI images were all slice time corrected, registered to the minimum outlier volume, and warped using non-linear transformations to MNI space. Volumes with motion greater than 0.3 mm (enorm) were censored, along with volumes where more than 5% of the voxels were calculated to be outliers (outlier fraction of 0.05), and the 6 head motion parameters were also regressed from the data. The four task conditions were included as regressors of interest. Smoothing was applied with a kernel size of 4 mm full width half maximum.

**Resting state fMRI.** The resting state pipeline followed the suggested steps for a comprehensive resting state analysis as outlined in the afni_proc.py documentation (example 11). Similar to the task data, the EPI images underwent slice time correction, registration to the minimum outlier volume, warping to the MNI template using non-linear transformations, censoring with a threshold of 0.3 mm (enorm) and an outlier fraction of 0.05, regression of the 6 head motion parameters, and smoothed with a kernel size of 4 mm FWHM. Additionally, the resting state data was despiked, and the derivatives of the 6 head motion parameters were also included in the regression, along with the first three principal components from the lateral ventricles, and the signal from the white matter using ANATICOR [3]. FreeSurfer was used to obtain the segmentations of the ventricles and the white matter (version 7.2.0; Fischl, 2012).

***Emotional conflict task***

The in-scanner task was run on Presentation Software v. 17.2 (Neurobehavioral Systems Inc., Berkeley, CA). There was a total of 148 trials, and each trial was presented for 1000 ms. Jittered fixation intervals ranging from 3000-5000 ms were interspersed between each trial. Participants had to respond by identifying the facial expression of the stimulus while ignoring the overlaid word. “Happy” and “fearful” responses were indicated with either the index or middle fingers, and the response finger assignment was counterbalanced over emotion type. Sex of model in the stimulus picture and emotion type were also counterbalanced in the task.

**2. Supplementary results**

***No significant difference in behavioral response to high and low conflict resolution trials in PTSD patients.***

For accuracy, we found a significant main effect of current trial (*t*_240_ = -11.034; *p* > 0.0001), main effect of previous trial (*t*_240_ = -2.782; *p* = 0.006), and previous trial x current trial interaction, as described by the original study (*t*_240_ = 3.435; *p* = 0.0007). However, post-hoc tests revealed that this effect was driven by a significant difference between the two levels of congruent trials (t_240_ = 2.782;  *p* = 0.007; *p*-values adjusted using the Tukey method), where congruent trials that followed congruent trials had a significantly greater accuracy than congruent trials that followed incongruent trials. The difference between the two levels of incongruent trials was not significant (t_240_ = -2.076; *p* = 0.1639). The interaction effect is visualized in **Supplementary Figure S5**.

For reaction time, there was a significant main effect of current trial (*t*_10346_ = 16.815; *p* > 0.0001) and previous trial (*t*_10346_ = 3.199, *p* = 0.0014). There was no significant interaction effect for previous trial x current trial (*t*_10346_ = -1.347; *p* = 0.1781). Treatment group, AEA levels, PCL scores, and CAPS scores were not significant in any of the subsequent models for accuracy or reaction time.

We also ran a linear mixed effects model on the neuroimaging task data without any of the covariates of interest to discover whether the previous trial x current trial interaction was significant in any clusters of the brain. There were 9 clusters where the effect of the interaction was significant, which were located in the bilateral anterior insula, the bilateral inferior frontal gyrus, the bilateral supplementary motor area (SMA) and in the right head of the caudate nucleus (**Supplementary Figure S6**). In all clusters except for the right caudate, there was greater activation for incongruent trials that followed congruent trials compared to incongruent trials that followed incongruent trials, similar to the findings in the original study [5]. In the right caudate cluster, this relationship was reversed. There were no significant clusters for the post-hoc *t*-test comparing the two congruent conditions. Detailed cluster information can be found in **Supplementary Table S3**.

**2. Supplementary tables**

| **Supplementary Table S1.** Adverse events. | | | | |
| --- | --- | --- | --- | --- |
|  | **Overall**  n=100 | **PBO**  n=49 | **FAAHi**  n=51 |  |
|  | **n (%)** | **n (%)** | **n (%)** | **p-value^1^** |
| Any AE | 84 (84%) | 42 (85.7%) | 42 (82.4%) | 0.65 |
| Any SAE | 0 | 2 (4.1%) | 0 | 0.24 |
| Any AE leading to withdrawal | 0 | 1 (2.0%) | 0 | 0.49 |
| **Adverse events, by preferred term^2^** | | | |  |
| Fatigue | 31 (31.0%) | 13 (26.5%) | 18 (35.3%) | 0.27 |
| Headache | 28 (28.0%) | 14 (28.6%) | 14 (27.5%) | 1.0 |
| Dizziness | 11 (11.0%) | 4 (8.2%) | 7 (13.7%) | 0.37 |
| Anxiety | 11 (11.0%) | 5 (10.2%) | 6 (11.8%) | 0.76 |
| Nasopharyngitis | 10 (10.0%) | 4 (8.2%) | 6 (11.8%) | 0.53 |
| Depressed mood | 8 (8.0%) | 3 (6.1%) | 5 (9.8%) | 0.49 |
| Nightmare | 8 (8.0%) | 3 (6.1%) | 5 (9.8%) | 0.49 |
| Insomnia | 9 (9.0%) | 5 (10.2%) | 4 (7.8%) | 1.0 |
| Dry mouth | 8 (8.0%) | 4 (8.2%) | 4 (7.8%) | 1.0 |
| Abdominal pain | 6 (6.0%) | 2 (4.1%) | 4 (7.8%) | 0.44 |
| Unintentional weight gain | 6 (6.0%) | 2 (4.1%) | 4 (7.8%) | 0.44 |
| Constipation | 5 (5.0%) | 1 (2.0%) | 4 (7.8%) | 0.2 |
| Decreased appetite | 6 (6.0%) | 3 (6.1%) | 3 (5.9%) | 1.0 |
| Influenza | 4 (4.0%) | 1 (2.0%) | 3 (5.9%) | 0.36 |
| Disturbance of attention | 3 (3.0%) | 0 (0.0%) | 3 (5.9%) | 0.11 |
| Paraesthesia | 6 (6.0%) | 4 (8.2%) | 2 (3.9%) | 0.69 |
| Covid-19 | 5 (5.0%) | 3 (6.1%) | 2 (3.9%) | 1.0 |
| Menstruation irregular | 5 (5.0%) | 3 (6.1%) | 2 (3.9%) | 1.0 |
| Nausea | 7 (7.0%) | 6 (12.2%) | 1 (2.0%) | 0.12 |
| Sleep disorder | 4 (4.0%) | 3 (6.1%) | 1 (2.0%) | 0.62 |
| Memory impairment | 4 (4.0%) | 4 (8.2%) | 0 (0.0%) | 0.12 |
| Blood potassium decreased | 3 (3.0%) | 3 (6.1%) | 0 (0.0%) | 0.25 |
| Libido decreased | 3 (3.0%) | 3 (6.1%) | 0 (0.0%) | 0.25 |
| ^1^ Chi-square (Pearson or Fischer’s exact test).  ^2^ Occurring in ≥5% of participants in any treatment arm. | | | | |

**Supplementary Table S2. Details regarding index traumas in the sample by treatment group in entire clinical trial sample.** Reprinted from Mayo et al. (2025) [6].

|  | **Overall**  N = 100 | **PBO**  N = 49 | **FAAHi**  N = 51 | **p-value**^1^ |
| --- | --- | --- | --- | --- |
| PTSD exposure type (CAPS-5) |  |  |  |  |
| Life threatening | 56 (56%) | 24 (49%) | 32 (63%) | 0.12 |
| Serious injury | 38 (38%) | 17 (35%) | 21 (41%) | 0.48 |
| Sexual assault | 52 (52%) | 29 (59%) | 23 (45%) | 0.36 |

**Supplementary Table S3. Significant clusters for the previous trial x current trial interaction across the whole sample.** Reported statistics and coordinates are of the cluster peak. MNI coordinates are reported in LPI orientation.

| **Cluster size** | **Peak *χ*^2^** | **x** | **y** | **z** | **Peak location** |
| --- | --- | --- | --- | --- | --- |
| 59 voxels | 24.084 | 31 | 25 | 1 | Right anterior insula |
| 21 voxels | 25.948 | 43 | 22 | 25 | Right inferior frontal gyrus (pars triangularis) |
| 20 voxels | 22.281 | 46 | 25 | 10 | Right inferior frontal gyrus (pars triangularis) |
| 19 voxels | 21.269 | 7 | 19 | 1 | Right thalamus |
| 19 voxels | 19.291 | -41 | 19 | 28 | Left inferior frontal gyrus (pars triangularis) |
| 18 voxels | 31.462 | 4 | 10 | 55 | Right supplementary motor area |
| 16 voxels | 18.541 | -8 | 7 | 55 | Left supplementary motor area |
| 13 voxels | 21.432 | -35 | 19 | -2 | Left anterior insula |
| 13 voxels | 19.537 | -41 | -2 | 37 | Left precentral gyrus |

**Supplementary Table S4. Clusters where PCL scores are significantly associated with vmPFC functional connectivity.** Reported statistics and coordinates are of the cluster peak. MNI coordinates are reported in LPI orientation.

| **Cluster size** | **Peak *β*** | **Peak *F*_1,72_** | **Peak *t*_72_** | **x** | **y** | **z** | **Peak location** |
| --- | --- | --- | --- | --- | --- | --- | --- |
| 100 voxels | 0.005 | 19.247 | 4.387 | -41 | 13 | 1 | Left anterior insula |
| 54 voxels | 0.005 | 13.138 | 3.625 | 64 | -17 | 16 | Right postcentral/supramarginal gyrus |
| 37 voxels | 0.005 | 18.783 | 4.334 | 34 | 7 | 13 | Right anterior insula |
| 36 voxels | 0.004 | 19.434 | 4.409 | -14 | -77 | 7 | Left cuneus |
| 36 voxels | 0.005 | 15.781 | 3.973 | -50 | -26 | 10 | Left posterior insula |
| 33 voxels | 0.005 | 16.686 | 4.085 | 19 | -53 | 73 | Right superior parietal lobule |
| 23 voxels | 0.006 | 21.112 | 4.595 | -62 | 1 | 4 | Left Rolandic operculum |
| 20 voxels | 0.005 | 13.841 | 3.720 | 58 | 7 | 4 | Right Rolandic operculum/precentral gyrus |
| 20 voxels | 0.004 | 11.830 | 3.440 | -44 | -38 | 49 | Left postcentral gyrus |
| 18 voxels | 0.005 | 12.134 | 3.483 | -2 | 1 | 49 | Right supplementary motor area |
| 17 voxels | 0.005 | 11.581 | 3.403 | -5 | -53 | 61 | Left precuneus |
| 13 voxels | 0.004 | 16.663 | 4.082 | -38 | -65 | -59 | Left cerebellum |
| 13 voxels | 0.004 | 15.104 | 3.886 | -17 | -41 | 46 | Left middle cingulate cortex |
| 12 voxels | 0.004 | 10.730 | 3.276 | 16 | -68 | 7 | Right primary visual cortex |
| 12 voxels | 0.005 | 17.687 | 4.206 | 49 | -29 | 52 | Right postcentral gyrus |
| 11 voxels | 0.004 | 22.751 | 4.770 | 34 | -17 | -2 | Right claustrum |
| 11 voxels | 0.003 | 14.317 | 3.784 | 13 | -23 | 7 | Right thalamus |
| 11 voxels | 0.005 | 15.538 | 3.942 | -62 | -14 | 10 | Left auditory cortex |

**Supplementary Table S5. Clusters where PCL scores are significantly associated with amygdala functional connectivity.** Reported statistics and coordinates are of the cluster peak. MNI coordinates are reported in LPI orientation.

| **Cluster size** | **Peak *β*** | **Peak *F*_1,72_** | **Peak *t*_72_** | **x** | **y** | **z** | **Peak location** |
| --- | --- | --- | --- | --- | --- | --- | --- |
| 158 voxels | 0.005 | 14.895 | 3.859 | 64 | -2 | 22 | Right Rolandic operculum/precentral gyrus |
| 120 voxels | 0.005 | 20.906 | 4.572 | -47 | -14 | 55 | Left precentral gyrus |
| 96 voxels | 0.005 | 19.338 | 4.397 | -41 | -47 | 49 | Left intraparietal sulcus |
| 32 voxels | 0.003 | 16.159 | 4.020 | -26 | 16 | 46 | Left middle frontal gyrus/premotor cortex |
| 22 voxels | 0.003 | 27.348 | 5.230 | -32 | 40 | 25 | Left middle frontal gyrus (rostral) |
| 21 voxels | 0.004 | 15.066 | 3.881 | -41 | 13 | 4 | Left anterior insula |
| 21 voxels | 0.004 | 17.300 | 4.159 | -5 | -8 | 58 | Left supplementary motor area |
| 20 voxels | 0.004 | 19.667 | 4.435 | -35 | -29 | 52 | Left postcentral gyrus |
| 15 voxels | 0.003 | 18.644 | 4.318 | -38 | 43 | 16 | Left dorsolateral prefrontal cortex |
| 15 voxels | 0.003 | 12.367 | 3.517 | 25 | -2 | 52 | Left frontal eye field |
| 14 voxels | 0.005 | 20.259 | 4.501 | -62 | 4 | 4 | Left Rolandic operculum |
| 12 voxels | 0.005 | 15.182 | 3.896 | -23 | -71 | 7 | Left primary visual cortex |
| 12 voxels | 0.004 | 13.295 | 3.646 | 52 | -26 | 55 | Right postcentral gyrus |
| 11 voxels | 0.004 | 13.249 | 3.634 | 49 | -56 | -14 | Right fusiform face area |

**3. Supplementary figures**

**Supplementary Figure S1. CONSORT chart.**

**
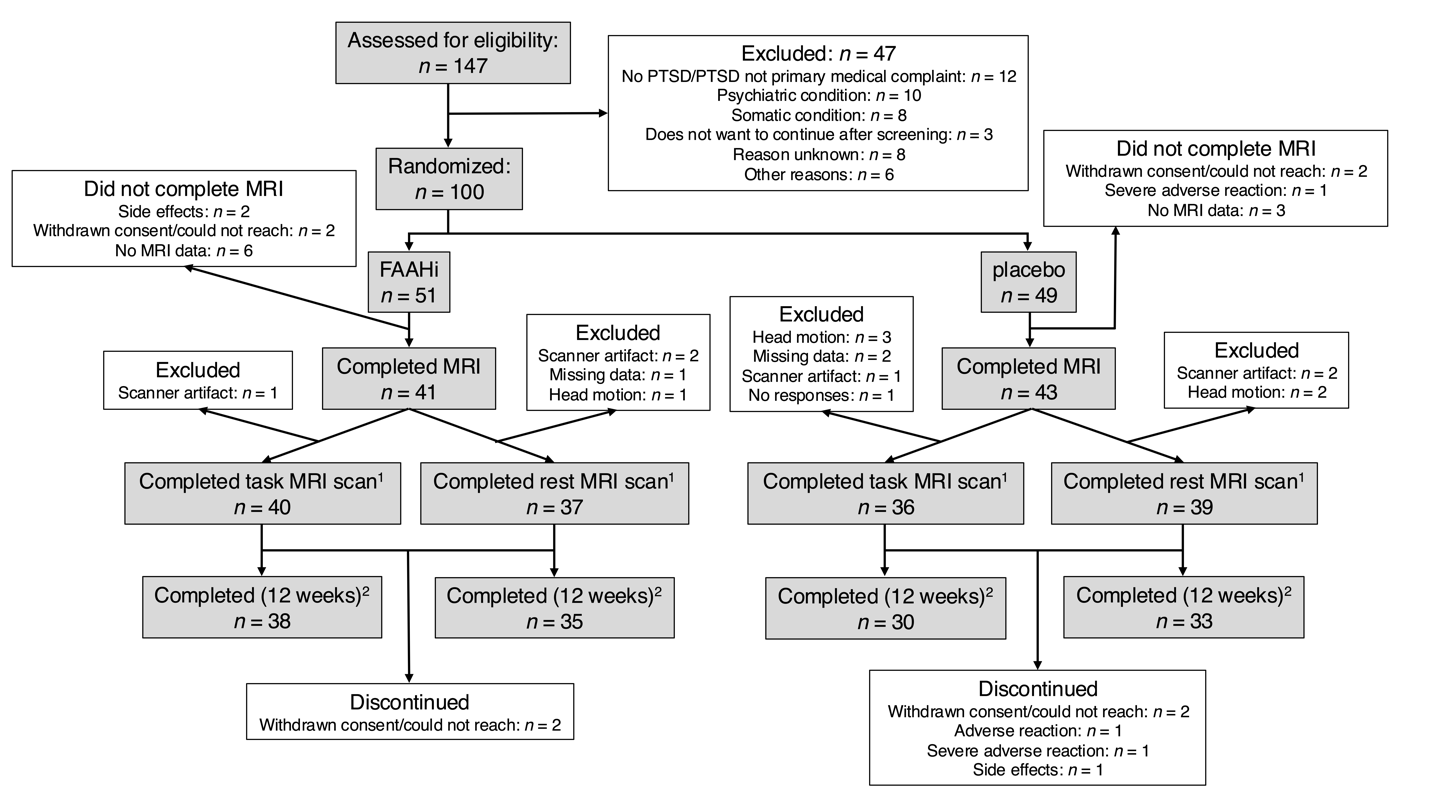
**

^1^Included in the PCL-5 analysis; ^2^Included in the CAPS-5 analysis.

**Supplementary Figure S2. Peripheral AEA concentrations throughout the trial.**

**
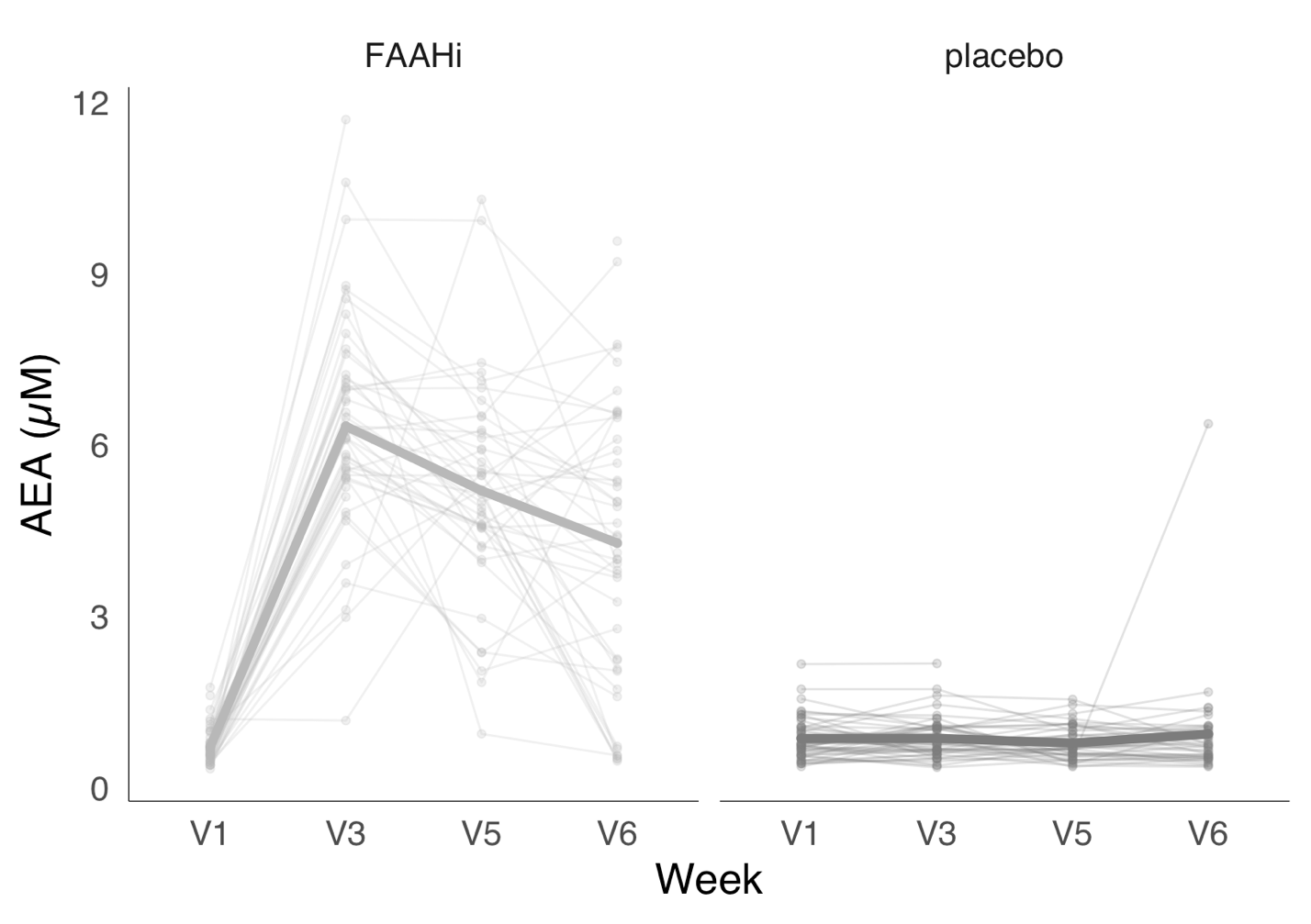
**

**Supplementary Figure S3. CAPS scores measured at baseline and endpoint of the trial.**

**
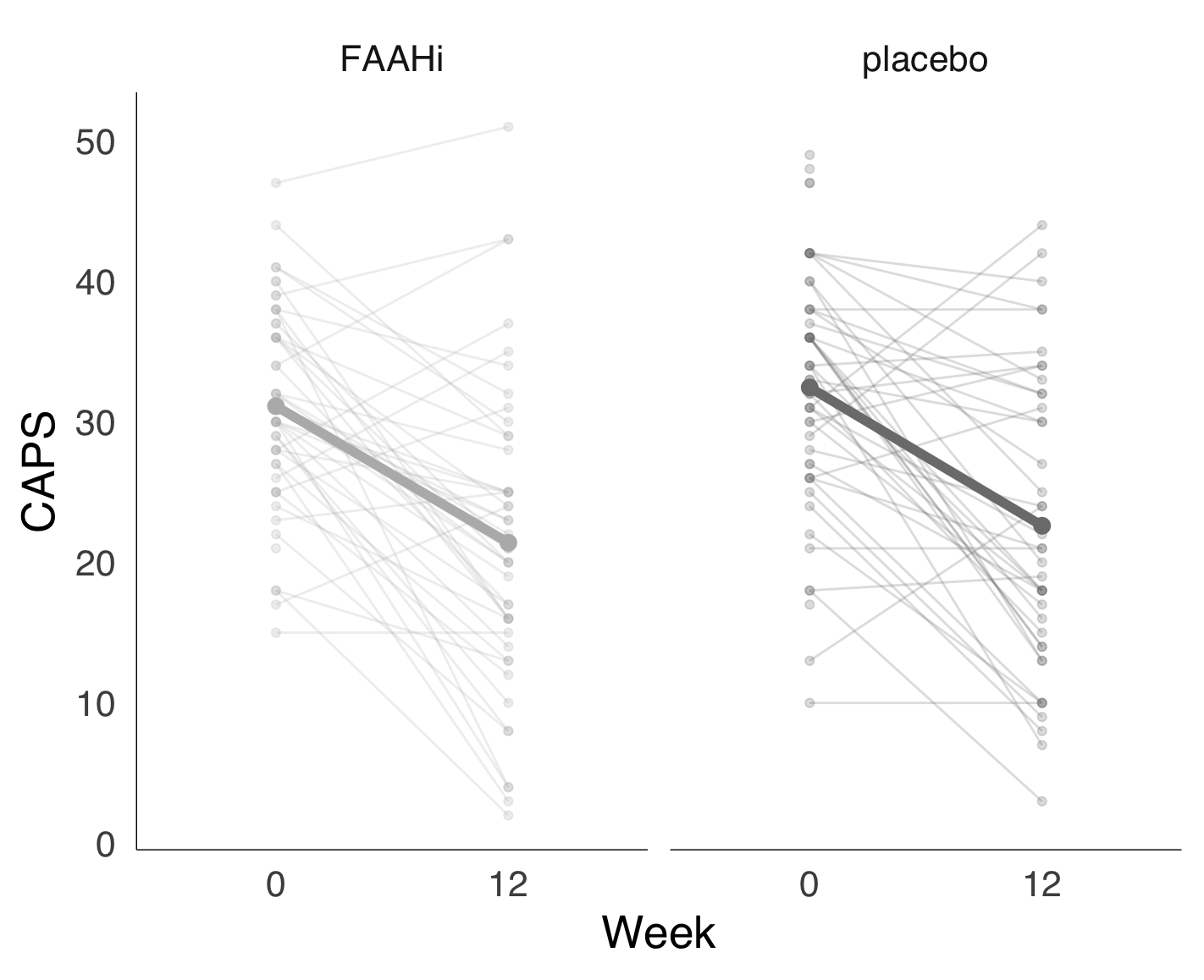
**

**Supplementary Figure S4. PCL scores measured throughout the clinical trial.**

**
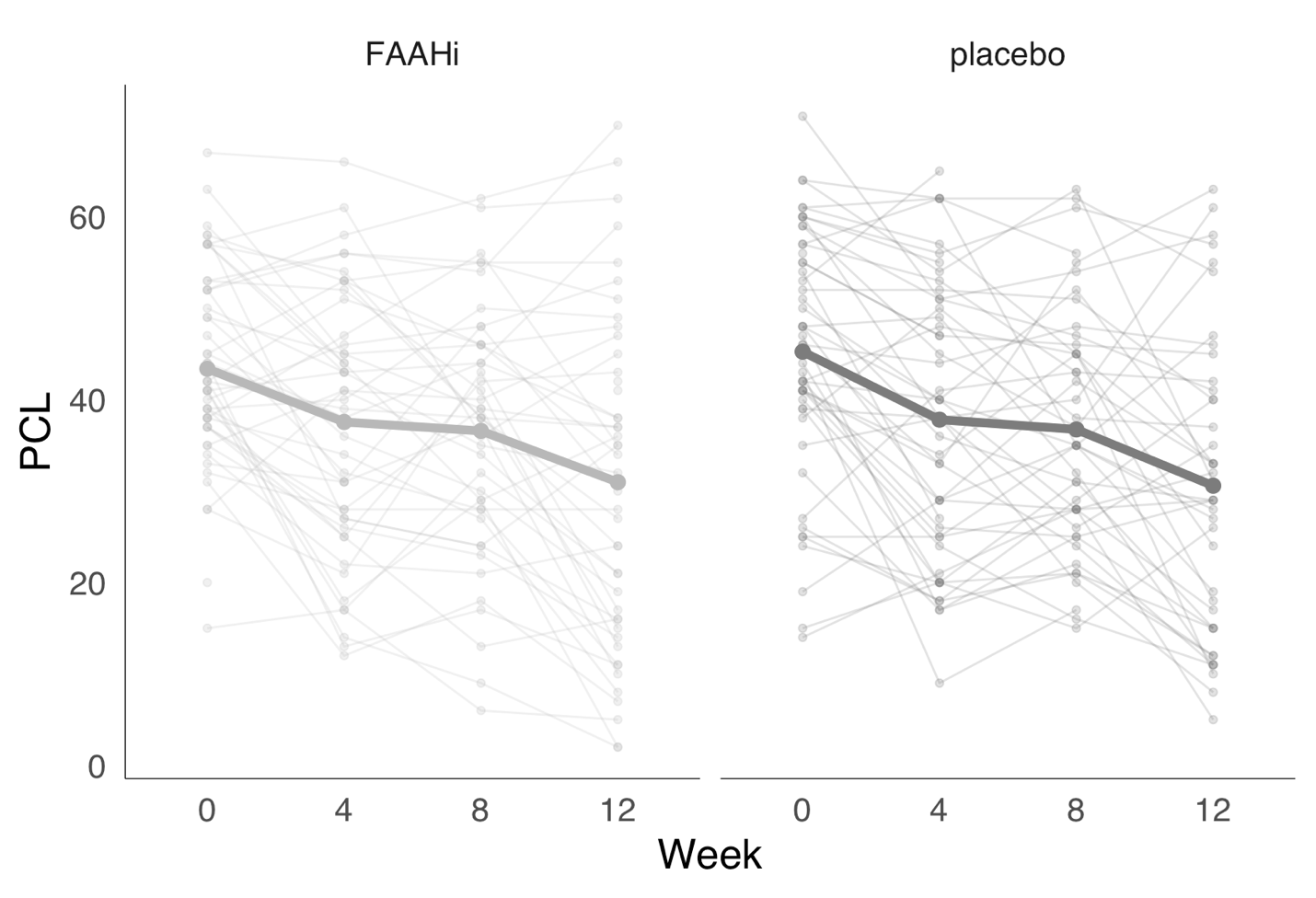
**

**Supplementary Figure S5. Behavioural data: previous trial x current trial interaction for accuracy.**


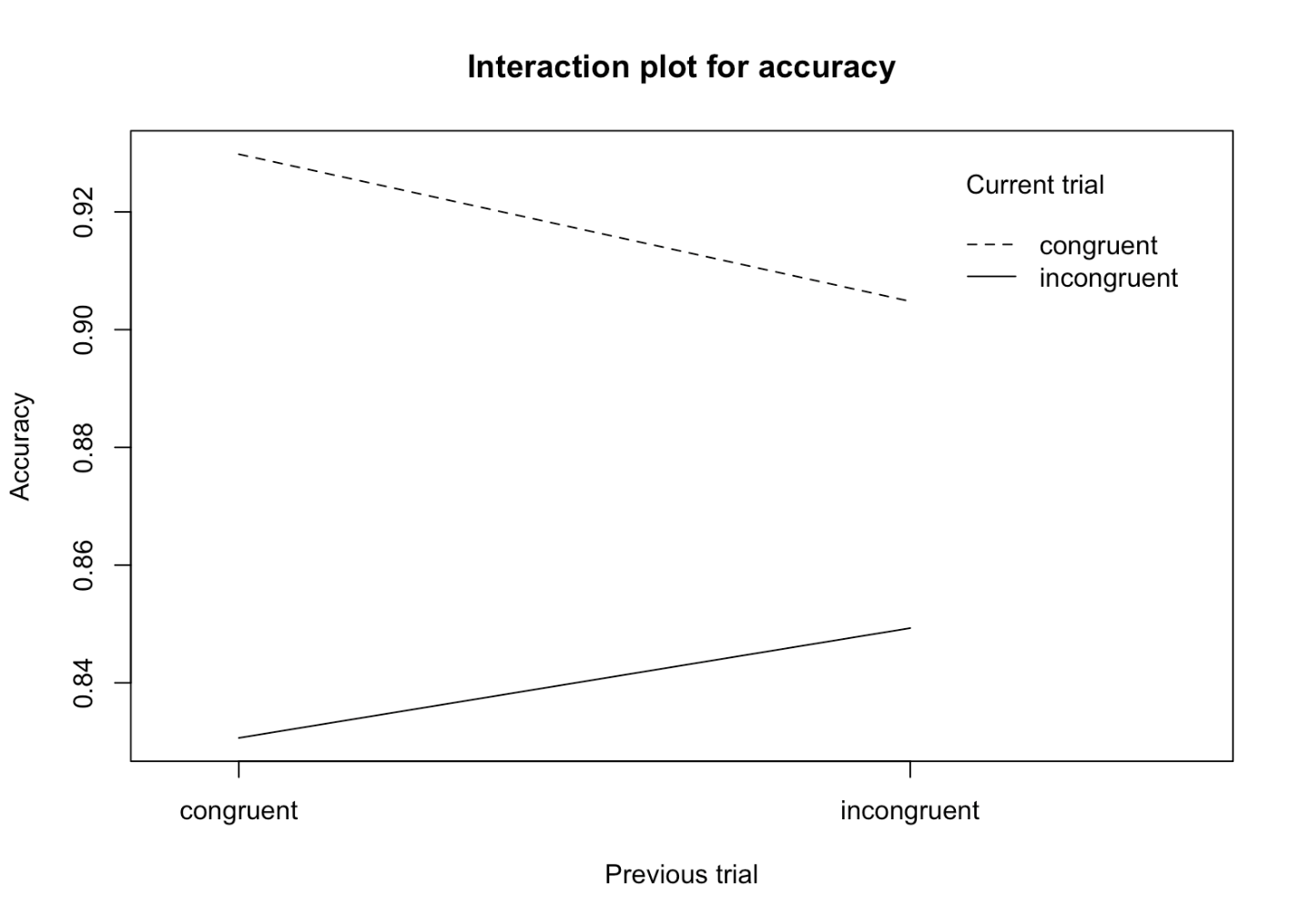


**Supplementary Figure S6. Neuroimaging data: previous trial x current trial interaction significant clusters.** Both colour bars represent the *Z*-statistic for the contrast of incongruent trials following congruent trials – incongruent trials following incongruent trials. For detailed cluster information, see **Supplementary Table S1.**


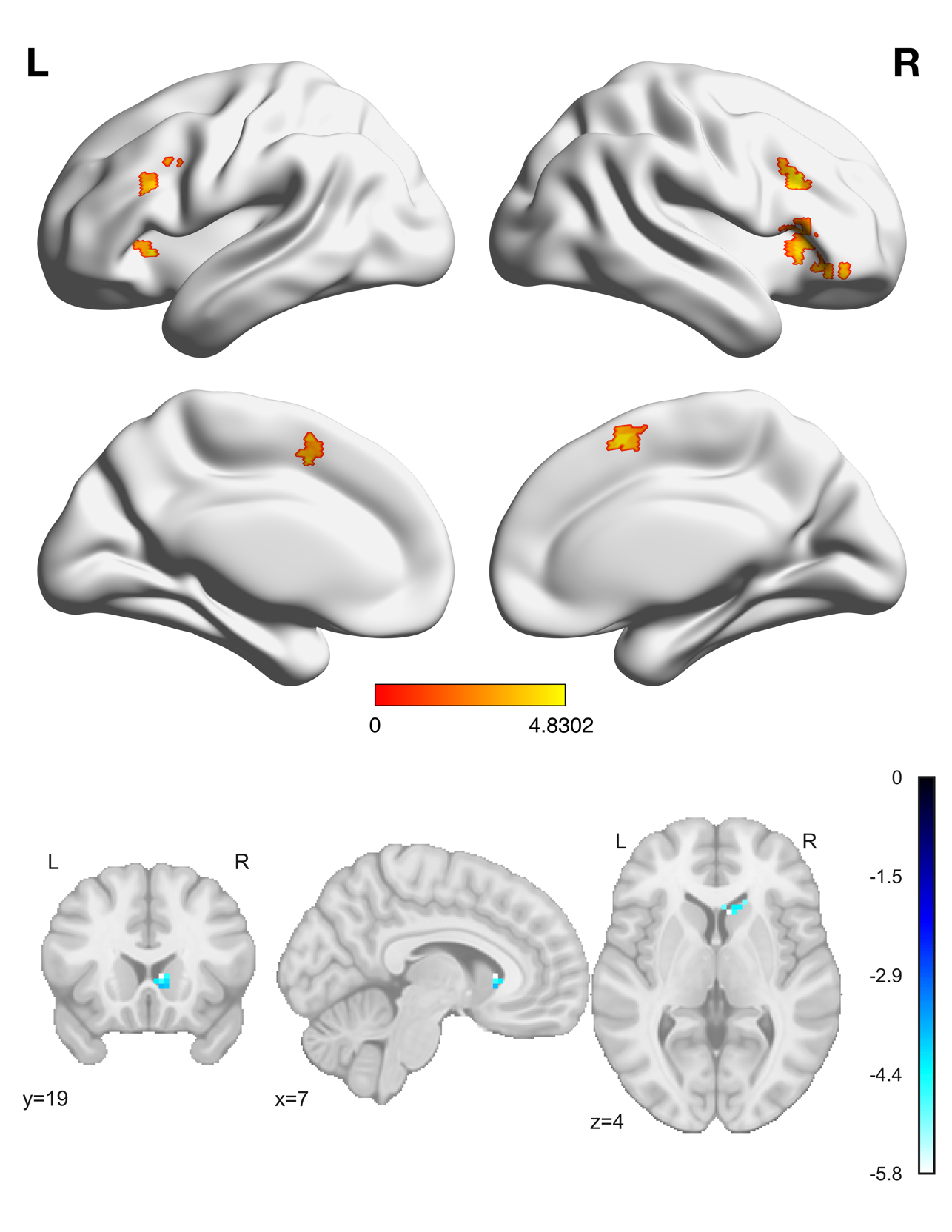


**References**

1. Marx BP, Lee DJ, Norman SB, Bovin MJ, Sloan DM, Weathers FW, et al. Reliable and clinically significant change in the clinician-administered PTSD Scale for DSM-5 and PTSD Checklist for DSM-5 among male veterans. Psychological Assessment. 2022;34:197–203.

2. Forkus SR, Raudales AM, Rafiuddin HS, Weiss NH, Messman BA, Contractor AA. The Posttraumatic Stress Disorder (PTSD) Checklist for DSM–5: A systematic review of existing psychometric evidence. Clinical Psychology: Science and Practice. 2023;30:110–121.

3. Jo HJ, Saad ZS, Simmons WK, Milbury LA, Cox RW. Mapping sources of correlation in resting state FMRI, with artifact detection and removal. NeuroImage. 2010;52:571–582.

4. Fischl B. FreeSurfer. NeuroImage. 2012;62:774–781.

5. Etkin A, Egner T, Peraza DM, Kandel ER, Hirsch J. Resolving Emotional Conflict: A Role for the Rostral Anterior Cingulate Cortex in Modulating Activity in the Amygdala. Neuron. 2006;51:871–882.

6. Mayo LM, Gauffin E, Petrie GN, Tansey R, Mazurka R, Haggarty CJ, et al. The efficacy of elevating anandamide via inhibition of fatty acid amide hydrolase (FAAH) combined with internet-delivered cognitive behavioral therapy in the treatment of post-traumatic stress disorder: a randomized, placebo-controlled clinical trial. Neuropsychopharmacol. 2025:1–9.
